# Factors Influencing Compliance with COVID-19 Health Measures: A Spanish Study to Improve Adherence Campaigns

**DOI:** 10.3390/ijerph19084853

**Published:** 2022-04-16

**Authors:** Nuria Galende, Iratxe Redondo, Maria Dosil-Santamaria, Naiara Ozamiz-Etxebarria

**Affiliations:** 1 Department of Developmental and Educational Psychology, Universidad del País Vasco/Euskal Herriko Unibertsitatea/University of the Basque Country UPV/EHU, 48940 Leioa, Spain; nuria.galende@ehu.eus (N.G.); iratxe.redondo@ehu.eus (I.R.); 2 Department of Research and Diagnostic Methods in Education, Universidad del País Vasco/Euskal Herriko Unibertsitatea/University of the Basque Country UPV/EHU, 48940 Leioa, Spain; maria.dosil@ehu.eus

**Keywords:** pandemic, compliance factors, age, sex, information campaigns, prevention

## Abstract

Since the spread of the COVID-19 virus was declared a pandemic, different measures have been taken to control it, including frequent hand-washing, the use of face masks and social distancing. Given the importance of these measures, the present study aims to assess compliance with them in a Spanish sample of 722 people aged between 18 and 65 years. It also aims to determine which factors influence the levels of compliance observed. Participants complied more with the rules in the public spaces. The younger group had lower levels of compliance than the older group. No differences were found in accordance with sex. It was shown that overall, the agents that most influenced compliance were family, testimonials and friends and fines. Some differences were observed in relation to age, and significant sex differences were found in some of these factors, with women scoring higher than men. The results are discussed in terms of their usefulness for the design of information campaigns that seek to foster a greater degree of engagement by the entire population and, ultimately, greater control of the pandemic, in addition to serving as a basis for the early prevention of the spread of new viruses in the future.

## 1. Introduction

### 1.1. COVID-19 Situation in Spain and Other Countries

In March 2020, the coronavirus disease 2019 (COVID-19; severe acute respiratory syndrome coronavirus 2, SARS-CoV-2) was recognized as a pandemic [1]. One month after that, the virus had infected over 556,864 people, and had resulted in over 44,090 deaths worldwide [2]. At the time of this article’s writing (March 2022), these figures have increased to 470,223,960 infected people and 6,094,326 deaths worldwide [3]. In the case of Spain, 11,451,676 people have been infected since the beginning of the pandemic, and 102,392 have died [4].

The rapid advance and high mortality rate associated with COVID-19 right from the beginning prompted governments to take drastic measures, including strict lockdowns [5] that were relaxed as infections, deaths and pressure on healthcare systems decreased [6]. Rapidly, several vaccines were approved and implemented, which helped considerably in changing the situation, at least in Europe. However, until the majority of the population was immunized, the behavioral measures implemented in most countries were still of vital importance at that point. That is the case of Spain, the country where this research was carried out.

In fact, the magnitude of this crisis was unprecedented in Spain. After Italy, it was the second-most affected European country at the beginning of the pandemic [7]. At some points, it became even the second country in incidence worldwide. Hence, there had to be implemented some of the strictest measures in Europe [8], including a strict lockdown that extended until May 2020. Then, when data started to improve, the government eased the measures [9], because, in addition to public health, the economic situation had to be taken into account. This study took place at that specific moment: a time when confinement was not so severe (it was reduced to a few hours at night) and other measures were adopted to return to social contacts, while still trying to maintain protection against the virus. More specifically, the measures implemented in Spain were the use of face masks, frequent hand-washing and social distancing.

These measures proved to be the most useful in attempting to curb infection until vaccines were available to ensure immunity to the virus. However, the success of the measures also depended on the level of compliance with them, because, although valuable, their usefulness disappeared the moment someone failed to comply with them. That is, the success of these government measures depended heavily on the willingness of the population to engage and comply with them [10]. Here, however, considerable differences emerged, and adherence to the guidelines was highly variable [11,12].

### 1.2. Sociodemographic and Individual Variables

As for the factors that influence compliance or non-compliance with the measures, studies point to a wide range of variables. Indeed, as a review of studies showed [13], factors such as being young and being male were associated with lower compliance, as were having an antisocial personality pattern, low empathy levels, low self-control and a tendency to engage in risky behaviors. The level of information to which individuals were exposed and the number of elderly people they knew also seemed to play a role. Some of these factors are presented in greater detail below.

In this sense, authors such as Margraf et al. [14] pointed out that both in Spain and in other countries young people show less adherence to rules, and Gutiérrez et al. [15], in the same line, found that the age groups of 20–30 years and below 20 were those who broke the rules the most. In countries such as France, Switzerland and Spain, men have been found less likely to follow the guidelines to contain spread of the virus [15,16,17]. In relation to personal variables, studies show that, unlike empathic people, those with antisocial traits are much less compliant with the rules [18,19,20]; in other words, those who are concerned and empathetic towards the most vulnerable engage in behaviors that protect them [21]. On the other hand, self-control is another important variable. Thus, Xu and Cheng [22] found that people with a high level of self-control are more compliant, and this variable remains significant even after controlling for other factors such as political ideology or demographic variables. The tendency to avoid risks has been also related to a greater compliance with measures such as social distance or the use of masks both in Spain and abroad [22,23]. Likewise, it has also been observed that the number of elderly persons people knew personally has an positive influence in the in compliance with standards as social distancing [24].

### 1.3. Other More General Influencing Variables

However, beyond personal and specific variables, it is interesting to focus on the influence of others that affect a larger number of people, since preventive programs can be better oriented along these lines.

Thus, for example, in a study carried out in Spain with general population, it was found that adequate knowledge was one of the determining variables for compliance with the three measures, along with attitudes and risk perception [25].

Trust in political leaders [26], as well as in the messages or information they transmit [27], has a significant influence on the level of compliance with the rules they impose. In addition, it seems advisable that the information is transmitted with certainty and based on solid evidence [28].

Furthermore, in research that studied the factors associated with compliance with social distance in Spain [29], it is suggested that in the future, increasing people’s trust in official information would be necessary. That way, it could reduce the influence of disinformation or even the belief in conspiracy theories, because both increase disobedience of this rule. This is consistent with what other authors have found in a positive sense [27] and consists of the fact that trust in the information transmitted by the government increases compliance with the rules and vice versa: one of the reasons why people sometimes do not comply with them is because they distrust the information that their government transmits.

The way in which messages are transmitted, and not only the level of confidence with which they communicate to the population, is also an important factor. For example, inducing emotions such as fear or guilt, as some advertising campaigns featuring very powerful real-life testimonials aim to do, can be a double-edged sword. For example, some authors have found that messages arousing fear are only effective when individuals feel that they are able to address the threat. In other words, such messages work as long as the fear induced does not become something inescapable or [30] for which they have no response (helplessness); otherwise, the responses activated are usually defensive in nature [31]. This occurs also with messages aimed at inducing guilt or shame in those who do not comply with the norms, as such messages tend to trigger mechanisms of moral disengagement [32].

In the case of adolescents and young people, the way the important information is communicated and who communicates to them is even more relevant [33]. Numerous studies have pointed out the impact that influencers have on young people with regard to the information they convey about COVID-19 compliance and other related issues [34].

Coercive strategies based on the punishment of undesired behaviors (fines, sanctions, etc.) seem also to have a questionable effect and depend on how recipients perceive this level of demand. In general, they can provide important motivation for complying with regulations, and some studies have found that youths who claimed to be socially distancing due to governmental sanctions or parental rules were also more engaged in this protective practice [35]. However, other studies have found that when rules are perceived as very strict, individuals report lower compliance, probably because respondents legitimize violating them by toning down their importance [36].

Finally, the influence of those nearest and dearest can be considered a key factor in compliance with the established norms. This has been observed in research carried out with the entire population, in which family and friends are viewed as having a strong influence [37]. Specifically, some studies focusing on young people have identified the desire to protect themselves and others, and even a feeling of social responsibility towards the community, as key reasons for complying with social distancing measures [30,35]. However, at this age, the influence of peers on the decision to engage (or not) in risky behaviors can be both positive and negative. For example, studies conducted in other contexts have shown that adolescents are more likely to experiment with tobacco, alcohol and drugs in the presence of peers [38] but are also less likely to engage in a risky behavior if a friend discourages them from doing so [39]. In general terms, young people are more socially influenced than adults to engage in prosocial behaviors [33], suggesting that the key would be to explore how, or through what strategies, peer influence could be harnessed in favor of compliance with protective measures.

In any case, it seems necessary to pay particular attention the factors that influence the young population as they are the ones who have most frequently skipped the measures or who have viewed the need to stay at home in the worst light [28]. Their idiosyncratic characteristics may need to be analyzed differentially and may also require differential intervention. Just as we can see that the impact on them is not the same as in the older population, perhaps the factors influencing their level of compliance are not the same either, and this is important when focusing information campaigns in the future.

In summary, when thinking about preventive strategies or awareness-raising campaigns, it is important to include interventions that are likely to have an impact on a large number of people. It is therefore necessary to determine which factors are important for the general population when it comes to conveying messages about compliance and determining which pathways are the most effective for this purpose and how exactly said messages should be framed. It is also important to think about how we can customize strategies by adapting them to different ages or profiles, since some studies indicate that this may be a key differential variable [40]. In this sense, although some issues were beginning to emerge quite clearly at the point our data were collected, there was still an urgent need for further research.

### 1.4. Justification for the Study

As stated earlier, few studies had focused on this field at the time this research was carried out, and many of those were conducted in cultural contexts that are very different from that in Spain. Given that the conception and understanding of rules and social customs can have a considerable influence on behavior, it is important to explore compliance levels with COVID-19 regulations in this specific context. For example, compliance with the norm regarding the use of face masks in a country such as Spain, where masks are not commonly used, may be different from that of countries where such habit is established, even in non-pandemic situations [41]. Although the results reported by other authors were interesting, specific research into compliance in this scenario was required.

On the other hand, most studies focus on studying the influence of one or very few variables on compliance, but it is of interest to simultaneously study the effect of several of them that may be relevant.

Furthermore, as noted, although many studies have focused on determining the factors involved in compliance in general, few studies have focused on investigating the differences between them in young people vs. in the adult population, or on possible sex differences with respect to these factors.

In light of the above, the present study had two aims: first, to assess the level of compliance with COVID-19 health regulations and explore possible sex and age-related differences; and second, to determine the people, factors and circumstances that may influence compliance with these regulations, again assessing possible age and sex-related differences.

The hypotheses of our study were therefore:

**Hypothesis** **1** **(H1).** *People comply more with the rules in public spaces*.

**Hypothesis** **2** **(H2).** *Men and younger people are less compliant with the established regulations*.

**Hypothesis** **3** **(H3).** *Family will be the most influential factor in compliance at all ages*.

**Hypothesis** **4** **(H4).** *Young people will place greater importance than adults on friends and influencers in compliance*.

**Hypothesis** **5** **(H5).** *Differences will be found between men and women with respect to the agents or circumstances that most influence them*.

## 2. Methods

### 2.1. Participants

The sample comprised 722 people, 73.5% women (*n* = 531), 25.8% men (*n* = 186) and 0.7% of non-binary sex (*n* = 5), aged between 18 and 65 years (*M* = 29.43, *SD* = 12.73). A large proportion of the sample hailed from the three provinces of the Basque Country (a region in northern Spain): 69.3% (*n* = 500) were from Bizkaia, 11.8% (*n* = 85) were from Gipuzkoa and 6.9%, (*n* = 50) were from Araba. The remaining 12% (*n* = 87) came from other provinces in Spain. In terms of education level, 49.4% of participants (*n* = 357) had university qualifications, 45.8% (*n* = 331) had a high school diploma, 3.7% (*n* = 27) had secondary level qualifications and 1% (*n* = 7) had primary level or no qualifications at all. In relation to certain COVID-19-related variables, the sample had the following characteristics: 7.8% (*n* = 56) had already had the disease; 29.4% (*n* = 212) had had close contact with someone who had had the disease and 30.3% (*n* = 219) had self-isolated for quarantine purposes at some point after the end of the general lockdown imposed on the entire population.

### 2.2. Materials

Participants completed the ad hoc questionnaires described below as part of a broader assessment package.

An ad hoc questionnaire was created to collect information on sociodemographic variables.

To assess the level of *compliance with COVID-19 health regulations,* participants were asked the following question: “During the past week, to what extent have you complied with the following COVID-19 prevention norms when you were in a social context (e.g., with friends)?” Items included “social distancing”, “face mask use” and “hand washing”, and responses were given on a 4-point scale (1 = not at all, 2 = a little, 3 = quite a lot, 4 = a lot).

To determine which *people, factors or circumstances influenced or facilitated their compliance,* participants were asked the following question: “On a scale from 1 (not at all) to 4 (very much), to what extent do you think the following people, factors or circumstances influence your level of compliance with the COVID-19 prevention rules?” The seven factors provided were: “your family”, “your friends”, “Youtubers, influencers, famous people”, “advertising campaigns”, “fines or penalties”, “testimonials by people who have had the disease” and “others” (in the last case, participants were asked to indicate any other influencing factors not included on the list provided).

### 2.3. Procedure

The study procedures were approved by the University of the Basque Country’s Research Ethics Board (M10/2020/070). A Google Forms questionnaire was created, and participants were contacted by email and cell phone, as well as through the social media, in October and November 2020 and asked to spread the word using the same means. Therefore, non-probability snowball sampling was used. The study was presented as an exploration of people’s perceptions of and agreement with COVID-19 regulations and some other peripheral factors, and participants were asked to give their consent before taking part in it. They were also informed that they could withdraw at any moment. Furthermore, all the requirements established in Organic Law 15/99 on Personal Data Protection were followed.

### 2.4. Data Analysis

Statistical analyses were performed with the IBM statistical package, SPSS Statistics for Windows, version 26 (IBM Corp., Armonk, NY, USA), calculating the sample size with the program *G Power.* First, the assumptions of normality and homoscedasticity of variances were checked to decide whether to use parametric or non-parametric tests. In the analysis of agents influencing compliance with the measures, a related sample was involved, and for this reason the critical level of *p* < 0.05 of the Shapiro–Wilks statistic was analyzed and found to be significant. Therefore, the Friedman test was used to test for differences between related groups. For differences between independent samples, the non-parametric Kruskall–Wallis test with Dunn’s post hoc was used. For this analysis, effect sizes were calculated according to Rosenthal and DiMatteo [42] and interpreted according to Cohen’s [43] test.

## 3. Results

### 3.1. Compliance with Measures in the Different Contexts with Friends, Family and Work

Compliance with the measures as a whole showed statistically significant differences according to context, *X*^2^*_F_* (2) = 759.25, *p* = 0.001. Likewise, between-group tests performed with Dunn’s post hoc tests showed significant differences between all contexts, with the average rank being higher at work or school (the place where most reported compliance with the measures), followed with friends and with family. However, Table 1 shows the differences of each measure as a function of the contexts. As can be seen, the mask shows statistically significant differences in all contexts, *X^2^_F_* (2) = 863.731, *p*= 0.001 with the average rank being higher at work or school, followed with friends and with finally. In the case of hand-washing, the statistic shows statistically significant differences, *X^2^_F_* (2) = 353.978, *p* = 0.001; however, the differences are only found in the at work or school; with family and at work or school; and with friends’ contexts. Finally, in the case of social distance, the differences are statistically significant, *X^2^_F_* (2) = 534.773, *p*= 0.001, and the pattern is the same as with the use of face masks, with differences in all contexts, at work or school again showing the highest average, followed with friends and with family.

### 3.2. Comparisons of Compliance with COVID-19 Health Regulations by Sex and Age

First, sex differences in compliance with health regulations (face mask use, hand-washing, social distancing and overall compliance) were explored. In terms of hand-washing, statistically significant sex differences were found, with women having a higher median than men, *U* = 42.871, *p* = 0.036, *r* = 0.22, with a small effect size. No sex differences were observed in relation to the other regulations: face mask use *U* = 46.977, *p* = ns, and social distancing *U* = 42.871, *p* = ns, or in terms of overall compliance *U* = 44.153.50, *p* = ns.

In relation to age, statistically significant differences were found in all the COVID-19 health regulations. Specifically, those aged 30 years and over had higher median for face mask use (*U* = 41.112.50, *p* = 0.001, *r* = 0.25), hand-washing (*U* = 41.683, *p* = 0.001, *r* = 0.26), social distancing (*U* = 25.845.50, *p* = 0.001, *r* = 0.20) and overall compliance (*U* = 31.147, *p* = 0.001, *r* = 0.22) than those aged under 30. In the case of face mask use and hand-washing, the effect size was intermediate; however, for social distancing and overall compliance was found a small effect size (see Table 2).

### 3.3. Descriptive Statistics for Influential People, Factors and Circumstances

The following Table 3 shows the statistically significant differences of the total sample among the agents that influence young people to comply with the health measures imposed to stop the spread of the pandemic, *X^2^_F_* (6) = 1.89.368.00, *p* = 0.001. Furthermore, between-group tests performed with Dunn’s post hoc tests showed significant differences between all agents except between the agents Youtubers, influencers and famous people (average range = 2.18) and politicians (average range = 2.50) and the groups fines or sanctions (average range = 4.46) and friends (average range = 4.52). These groups seem to be in the same range.

### 3.4. People, Factors or Circumstances by Age

We also analyzed differences between the two age groups in terms of the people, factors or circumstances that influenced compliance. Significant differences were found in relation to friends (*U* = 49.687.50, *p* = 0.001, *r* = 0.27), with those over 30 scoring higher than their younger counterparts; Youtubers, influencers and famous people (*U* = 52.653.50, *p* = 0.001, *r* = 0.28), with higher scores among those aged under 30; and fines or sanctions (*U* = 41.435.00 *p* = 0.001, *r* = 0.24), with higher scores again among those aged under 30. Friends and Youtubers, influencers and famous people had a small effect size, whereas fines and sanctions had an intermediate effect size (see Table 4).

### 3.5. Sex Differences in People, Factors or Circumstances Influencing Compliance by Age Group

Table 5 presents the descriptive statistics and sex differences in relation to the factors influencing compliance with COVID-19 regulations in the two age groups (<30 and >30 years). In the case of the younger age group (those aged between 18 and 30), family (*U =* 18.360.50, *p =* 0.033, *r =* 0.22), friends (*U =* 17.391.00, *p =* 0.04, *r =* 0.22), news (*U =* 17.256.50, *p =* 0.003, *r =* 0.22) and testimonials (*U =* 13.477.50, *p =* 0.001, *r =* 0.22) were the factors for which statistically significant sex differences were observed, all with small effect sizes. In the case of older participants (aged 30 and over), statistically significant sex differences were found in family (*U =* 4.810.50, *p =* 0.007, *r =* 0.22), friends (*U =* 4.863.50, *p =* 0.011, *r =* 0.22), politicians (*U =* 4.963.00, *p* = 0.020, *r =* 0.22) and testimonials (*U* = 5.045.50, *p* = 0.036, *r* = 0.22), all with a small effect size. In both age groups, women had higher medians than men in all the factors mentioned above, but all have a small effect size.

## 4. Discussion

This study aimed to explore differences in compliance with COVID-19 hygiene standards in terms of context, sex and age. It also attempted to determine which agents have the greatest influence on compliance with those hygiene norms in general and also with regard to sex and age.

The results of the present study reveal that, in general, people comply fairly well with the regulations designed to prevent the spread of COVID-19, a finding which is consistent with those reported by previous research [44]. In relation to the first hypothesis, as expected, the study revealed that the tendency to comply is stronger at work, weaker among friends and weaker still in the family environment. This is also the case when looking at each of the standards (mask use, hand-washing and social distance) separately. These results are understandable, as when schools reopened their doors after the lockdown, they were obliged to implement strict hygiene measures [45], and the same occurred when people returned to their jobs after teleworking [46], circumstances which most likely resulted in greater compliance with the established measures in these environments. In connection to compliance with the rules while with friends, it may be that people are somewhat less compliant in this environment, either because the encounters tend more to be outdoors (meaning that they consider the measures to be unnecessary) or because they are meeting in private places where fines and penalties do not apply. This is understandable up to a point, as socializing is a human need (and is especially relevant for those under 30 years of age), and its absence may generate symptoms of anxiety, depression and emotional disorders [47]. Nevertheless, and in view of the results, it seems necessary to place greater emphasis on the need to comply with the rules in this context, since it is probably an important source of transmission. Not surprisingly, the family environment is the context in which compliance is lowest, as it includes cohabiting people. In the case of non-cohabiting relatives, in general, people abide by the measures, even when these involve not seeing family members for long periods of time [48], with the consequent feelings of loneliness that this may generate [49]. In this sense, future research should try to determine to which type of relative (cohabiting or non-cohabiting) participants are referring in their answers, since non-compliance with the rules when with non-cohabiting family members may be especially dangerous in terms of the spread of the virus.

Following the second hypothesis, the differences observed in compliance levels were also analyzed in accordance with both sex and age. In terms of sex, differences were only found in the case of hand-washing, although with a small effect size, and unlike in some other studies [31], no differences were found in any of the other measures analyzed. This may be due to the overrepresentation of women in our sample, although it would be interesting to explore this result in more depth. In terms of age, statistically significant differences were found in all COVID-19 health measures, with those under 30 being the least compliant. It has previously been reported that younger people find it more difficult to comply with the COVID-19 regulations [13], especially the one referring to social distancing, due to the fact that those in this age group need to be close to peers at this vital stage of their development [50]. This finding is consistent with those reported in previous studies [14,16,44] and indicates that, although in general terms compliance rates are not bad, it seems that this age group functions differently.

In terms of the people and factors that may influence compliance with the health guidelines in the whole sample, the most influential were in order of highest to lowest family, real-life testimonials, friends and fines and penalties (at the same level), news, politicians and influencers (at the same level).

Finding that family has the strongest influence is not particularly surprising, and this result was in line with the third hypothesis. In fact, caregiving is one of the factors that studies show to play a major role in meeting the established standards [30,35]. Moreover, both young people and adults point to family as one of the aspects that most influence their behavior [37].

Real-life testimonials were shown to be the second-most important in the list of variables that the participants scored. This is interesting, as there has been much controversy in this regard, with some authors arguing that testimonials, if they are hard-hitting, not only fail to raise awareness, but often generate rejection [31]. However, in light of the responses obtained in the present study, it is likely that in our context, this type of message has been well managed, and this strategy should be maintained in future campaigns.

At the lower end of the list two groups are found: politicians and influencers. This was to be expected in the case of influencers, as they are agents that mainly tend to have an impact on young people [34], but not on people of all ages. Furthermore, social desirability may have a role here, as it is probably quite embarrassing to acknowledge that one acts under the influence of these figures, particularly in relation to an issue as serious as COVID-19. However, the fact that politicians also appear in this last place and at the same level as the previous ones, deserves some reflection. Thus, given that trust in political leaders is associated with greater compliance [26], we could interpret that the Spanish population does not trust these agents very much, which could detract from the credibility of their messages and therefore not translate into desirable behaviors.

In relation to the factors not on the initial list that were identified by participants, it should be noted that respondents attach particular importance to the information provided by experts and/or scientists, as well as to their own criteria, when it comes to complying with the measures. Regarding the first factor, governments should give experts a more prominent role when communicating measures to citizens. In relation to the second, although there are multiple factors or circumstances that can obviously influence our decision-making, the truth is that many people are aware of and comply with the measures as a result of their own conviction and social awareness. This finding is a very important one, since those who comply for these reasons are a major asset in controlling the virus, since they act with much more conviction.

On the other hand, when comparing the under-30 and older-age groups in terms of agents that influence them, it becomes apparent that fines and penalties have a greater influence on the younger age group. This may be due to the fact that, at this age, people’s behavior is still more often guided by extrinsic motivation (reward–punishment) and is based more on the achievement of immediate short-term results [32] than on social awareness. Moreover, studies have found that those who perceive rules as too strict or demanding tend to break them [36], something that may occur more frequently in this age group.

Contrary to what we expected in the fourth hypothesis, friends were found to have a greater influence in the older age group. This deserves further exploration, since all the literature points to the enormous impact of friends, especially on the behavior of young people and adolescents [38,39]. On the other hand, as hypothesized, Youtubers, influencers and famous people were significantly more important for compliance in the under-30 age group. This is in line with the literature, where the impact they have on young people and the capacity to mobilize them at a large scale is repeatedly pointed out [51,52]. Hence, some studies have shown that influencers have become especially crucial during a pandemic like COVID-19, when uncertainty was extreme, and people looked to key opinion leaders for information and guidance [34].

In relation to gender, and confirming our fifth hypothesis, many of the variables studied (family, friends, news and testimonials) were rated significantly higher by women than by men. Moreover, with the exception of testimonials, this pattern was observed in both age groups. This may be because men are more reluctant to admit that their behavior is influenced by the factors presented, or alternatively, it may be because other factors or motives that were not included in this study drive their behavior.

## 5. Conclusions

The present study provides some clues about the factors that must be taken into account when creating campaigns to take care of hygiene measures in the case of pandemics that may occur in the future.

In terms of compliance, the most frequent places to comply with the rules seems to be the workplace, followed by a friends-and-family environment. In this sense, it would be necessary to make more emphasis on the importance of following them also in intimate contexts, where contagions could easily spread because we feel illusorily more protected in them.

In terms of age groups, those under 30 years of age are the least compliant with the rules. This is why campaigns should be directed more towards them in the future, taking into account their specific motivations and needs. In this regard, it may help to give young people the option of participating in the design of preventive campaigns [32,33,53], so that they feel that their needs have been taken into account. This, in turn, would probably lead to a greater commitment to the health measures with which they have to comply. Likewise, and as the study shows, we should not forget the importance of the social media and the value young people and adolescents attach to the messages conveyed by those who appear in them. For example, it may be particularly useful to target social media [36] “influencers”, individuals with a strong online presence and a large number of adolescent followers. If these individuals model positive social distancing behavior and communicate the risk of COVID-19 through their platforms, adolescents may listen [33].

On the other hand, and in terms of the most influential agents in all age groups, family and real-life testimonials are found. Therefore, agents that are close to families should be involved in compliance campaigns, making them understand that they are the ones who will have the greatest influence on their family members. On the other hand, first-person testimonials could continue to be used in our context, since it is easy for people to empathize with them and their experiences, and they seem to have an impact that has a positive effect on their behavior. In addition, government and authorities may improve their credibility, giving transparent information and messages that point out both the purpose and the effectiveness of the required actions [14]. This is something to which they should pay special attention, as satisfaction with the information provided and the reaction of the government during the pandemic significantly predict perceptions of the efficacy and usefulness of the restrictions [54] and, consequently, motivation to comply with the rules [14].

The study has some limitations that should be taken into account in the future. First, the sample should have been larger and more balanced in terms of sex. Second, data collection through self-reports may introduce biases due to the influence of social desirability, something that should be controlled by implementing research strategies such as those suggested by some authors [55]. Furthermore, validated measures for collecting this type of data do not yet exist. Finally, the data collection format (online) may have influenced the characteristics of the sample obtained (e.g., overrepresentation of people who are more motivated and concerned about the pandemic). Besides, future studies should increase the sample of participants, also including more men to make the sample more representative, and further investigate the needs of different groups to improve compliance.

Nevertheless, and despite these limitations, the study highlights the idea that in future pandemics, more resources should be given to families to facilitate compliance. Masks, hydrogel and antigen testing should be made more economical and accessible in future pandemics. In addition, the need for people to get together with their friends and family should be taken into account and public outdoor areas should be made available for them to meet. In short, the study makes a major contribution to the literature on the subject, providing information on the factors that most influence compliance with regulations and offering relevant data for guiding prevention campaigns for both this pandemic and future situations of social emergency. This is extremely necessary, considering that it is very likely that in the future we will face again the situation we are currently experiencing or other similar situations. Knowing what most influences people to comply with the established health measures will be of vital importance in these possible scenarios in order to tackle the problem from the beginning and thus avoid a rapid spread of the virus before it becomes a global pandemic, as on this occasion.

## Figures and Tables

**Table 1:** The differences of each measure as a function of the contexts.

Use of Masks Contexts	Average Range	X^2^_F_* (p)	Post Hoc	Hand-Washing Contexts	Average Range	X^2^_F_* (p)	Post hoc	Distance Social Contexts	Average Range	X^2^_F_* (p)	Post Hoc
1. with friends	2.08	863.731 (0.001 ***)	1–2 2–3 3–1		1.80	353.978 (0.001 ***)	2–3 1–3		1.93	534.773 (0.001 ***)	1–2 2–3 3–1
2. with family	1.28				1.76				1.53		
3. at work or school	2.63				2.44				2.54		

* X^2^_F_ = Friedman statistics; *** *p* < 0.001

**Table 2:** Mean differences in compliance with COVID-19 health regulations by age.

Compliance–Age	Mdn (IIQ)	n	*U*	*p*	*r*
Face mask use			41.112.50	0.001 ***	0.25
<30	9 (8.66–8.95)	464			
>30	10(9.40–9.83)	247			
Hand-washing			41.683.00	0.001 ***	0.26
<30	9 (8.77–9.13)	464			
>30	10 (9.67–10.12)	247			
Social distancing			25.845.50	0.001 ***	0.20
<30	6 (6.29–6.60)	464			
>30	8 (8.12–8.59)	247			
Overall Compliance			31.147.00	0.001 ***	0.22
<30	24 (23.82–24.58)	464			
>30	28 (27.29–28.43)	247			

Note: *** *p* < 0.001; r = effect size.

**Table 3:** Descriptive statistics for influential people, factors and circumstances.

Influential People	Average Range	X^2^_F_* (p)	Post Hoc
1. Family	5.55	1.789.368.00 (0.001 ***)	1—2, 3, 4, 5, 6, 7 2- 1,3,5,6,7 3- 1,2,4,6,7 4- 1,3,5,6,7 5- 1,2,4,6,7 6- 1,2,3,4,5,7 7- 1,2,3,4,5,6
2. Friends	4.52		
3. Youtubers, influencers and famous people	2.18		
4. Fines or sanctions	4.46		
5. Politicians	2.50		
6. News	3.844		
7. Testimonials	4.94		

Note: * X^2^_F_ = Friedman statistics; *** *p* < 0.001.

**Table 4:** Mean differences in the people, factors or circumstances influencing compliance with COVID-19 regulations by age.

Factors–Age	Mdn (IIQ)	n	*U*	*p*	*r*
Family			59.342.00	0.976	-
18–30	4 (3.27–3.42)	466			
>30	4 (3.25–3.44)	255			
Friends			49.687.50	0.001 ***	0.27
18–30	3 (2.67–2.83)	466			
>30	3 (2.90–3.10)	255			
Youtubers, influencers and famous people			52.653.50	0.001 ***	0.28
18–30	1 (1.57–1.74)	466			
>30	1 (1.36–1.54)	255			
Fines or sanctions			41.435.00	0.001 ***	0.24
18–30	3 (2.90–3.08)	466			
>30	2 (2.30–2.55)	255			
Politicians			59.435.00	0.965	.
18–30	2 (1.71–1.88)	466			
>30	2 (1.65–1.85)	255			
News			56.606.50	0.249	
18–30	2.50 (2.38–2.56)	466			
>30	3 (2.42–2.65)	255			
Testimonials			59.391.00	0.952	
18–30	3 (2.94–3.09)	466			
>30	3.05 (2.88–3.11)	255			

Note: *** *p* < 0.001; r = effect size.

**Table 5:** Descriptive statistics and significant sex differences in compliance within the sample age groups.

<30 Age	Mdn (IIQ)	n	*U*	*p*	*r*	>30 Age	Mdn (IIQ)	n	*U*	*p*	*r*
Family			18.360.50	0.033 *	0.23	Family		189	4.810.50	0.007 **	0.18
Female	4 (3.32–3.48)	341				Female	4 (3.31–3.53)	64			
Male	3 (3.05–3.36)	122				Male	3 (2.91–3.34)				
Friends			17.391.00	0.004 **	0.22	Friends			4.863.50	0.011 *	0.18
Female	3 (2.73–2.91)	341				Female	3 (2.97–3.19)	189			
Male	3 (2.40–2.71)	122				Male	3 (2.58–2.99)	64			
Youtubers, influencers and famous people			19.853.50	0.573		Youtubers, influencers and famous people			5.652.50	0.338	
Female	1 (1.53–1.72)	341				Female	1 (1.31–1.51)	189			
Male	1 (1.57–1.94)	122				Male	1 (1.35–1.81)	64			
Fines or penalties			20.182.50	0.573		Fines or penalties			5.153.00	0.075	
Female	3 (2.93–3.13)	341				Female	2 (2.34–2.63)	189			
Male	3 (2.69–3.10)	122				Male	2 (1.95–2.48)	64			
Politicians			20.703.00	0.892		Politicians			4.963.00	0.020 *	0.19
Female	2 (1.70–190)	341				Female	2 (1.69–1.93)	189			
Male	2 (1.62–1.96)	122				Male	1 (1.37–1.79)	64			
News			17.256.50	0.003 **	0.22	News			5.321.00	0.124	
Female	3 (2.46–2.65)	341				Female	3 (2.46–2.72)	189			
Male	3 (2.09–2.42)	122				Male	3 (2.13–2.62)	64			
Testimonials			13.473.50	0.001 ***	0.24	Testimonials			5.045.50	0.036 *	0.20
Female	3 (3.10–3.26)	341				Female	3 (2.94–3.21)	189			
Male	3 (2.40–2.74)	122				Male	3 (2.56–3.04)	64			

Note: *** *p* < 0.001; ** *p* < 0.01; * *p* < 0.05; r = effect size.

## Data Availability

The data that support the findings of this study are available on request from the corresponding author.
